# UV-B antagonises shade avoidance and increases levels of the flavonoid quercetin in coriander (*Coriandrum sativum*)

**DOI:** 10.1038/s41598-017-18073-8

**Published:** 2017-12-19

**Authors:** Donald P. Fraser, Ashutosh Sharma, Taryn Fletcher, Simon Budge, Chris Moncrieff, Antony N. Dodd, Keara A. Franklin

**Affiliations:** 1 School of Biological Sciences, University of Bristol, Bristol, UK; 2 Vitacress Herbs, Chichester, West Sussex UK

## Abstract

Despite controlling a diverse array of regulatory processes in plants, UV-B wavelengths (280–315 nm) are attenuated by common greenhouse materials such as glass and polycarbonate and are therefore depleted in many commercial growing environments. In this study, we analysed the architecture, pigment accumulation and antioxidant capacity of coriander (*Coriandrum sativum*, also known as cilantro) plants grown with and without supplementary UV-B (1.5 µmol m^−2^ s^−1^). We demonstrate that UV-B limits stem elongation responses to neighbour proximity perception (shade avoidance), promoting a more compact plant architecture. In addition, UV-B increased leaf quercetin content and total antioxidant capacity. *Arabidopsis thaliana* mutants deficient in flavonoid biosynthesis were not impaired in shade avoidance inhibition, suggesting that UV-B-induced flavonoid synthesis is not a component of this response. Our results indicate that UV-B supplementation may provide a method to manipulate the architecture, flavour and nutritional content of potted herbs whilst reducing the deleterious impacts of dense planting on product quality.

## Introduction

UV-B comprises less than 1% of sunlight, yet regulates a wide range of physiological and developmental processes in plants. These include the production of protective sunscreen compounds, plant growth, photosynthesis, stomatal regulation and defence against pests and pathogens^1^. In Arabidopsis, photomorphogenic UV-B signals are perceived by the photoreceptor UV RESISTANCE LOCUS 8 (UVR8)^2^. Upon UV-B absorption, UVR8 dimers monomerise and interact with the E3 ubiquitin ligase CONSTITUTIVELY PHOTOMORPHOGENIC 1 (COP1) to initiate the transcriptional regulation of hundreds of genes^3^. The most striking phenotype displayed by UV-B-treated plants is a reduction in plant size, resulting from reduced leaf expansion and an inhibition of stem elongation^4,5^. This is most apparent in environmental conditions whereby stem elongation is promoted, such as exposure to low red to far red ratio light (low R:FR)^5^, and high temperature^6^. Low R:FR is a component of vegetational shade and a signal of neighbour proximity prior to shading^7^. Plant perception of reduced R:FR by phytochrome photoreceptors leads to a rapid elongation of stems, as plants attempt to overtop neighbours. This response is largely driven by an increase in auxin synthesis, via low R:FR-mediated stabilisation and activation of PHYTOCHROME INTERACTING FACTOR (PIF) transcription factors^7^. UV-B perceived by UVR8 decreases auxin activity by reducing PIF4/5 abundance and activity. This is achieved by promoting PIF degradation^5^, increasing levels of ELONGATED HYPOCOTYL 5 (HY5) transcription factor (which competes with PIF4 for target promoters)^8^ and increasing levels of growth-suppressing DELLA proteins which bind to PIFs, inhibiting their function and promoting their degradation^5,9,10^.

Control of stem length is a key objective in horticulture, where the aesthetic quality of plants is paramount to commercial success. Excessive stem growth can reduce both the market value and shelf life of produce. This problem is exacerbated by dense planting which can initiate shade avoidance responses^7^. The molecular processes controlling light-regulated stem elongation are well characterised in Arabidopsis, but our understanding of photomorphogenesis in culinary herbs is more limited. Recent studies have, however, shown that mixed narrow bandwidth LED treatments of red, blue, green and yellow light can be used to manipulate the plant architecture and antioxidant content of sweet basil and coriander^11,12^. Manipulation of UV-B signalling in commercial growing environments is an emerging area of research^13–16^. One study reports that lettuce pre-treated with UV-B outperformed control plants, post transplantation to field^17^. UV-B supplementation has also been shown to increase the leaf area, dry biomass and antioxidant capacity of sweet basil, thus providing a potential mechanism to adjust the harvest yield and nutritional content of horticultural produce^18^. In coriander, high dose narrowband UV-B has been shown to induce chromosomal aberrations, and decrease pigment and carbohydrate content^19^. The impact of lower dose (non-stressful) levels of UV-B on coriander growth and development remains poorly studied.

Here, we analysed the effects of low dose UV-B on coriander architecture, pigment and antioxidant content, in backgrounds of high and low R:FR, to simulate well-spaced and dense planting, respectively. We sought to determine whether coriander displays similar UV-B-mediated photomorphogenic responses to Arabidopsis^1,5^ to establish whether UV-B supplementation could, in principle, be incorporated into commercial lighting regimes to improve the quality of potted herbs.

## Materials and Methods

### Plant material

*Coriandrum sativum* cv. Slow Bolt and cv. Cruiser were provided by Vitacress Herbs Ltd. *Arabidopsis thaliana tt4* and *tt7* mutants in the Col-0 background^20^ were used for comparison in flavonol glycoside detection experiments and hypocotyl elongation assays.

### Growth conditions

*Coriandrum sativum* seeds were scarified to break dormancy and synchronise germination. Fruit were manually split into two mericarps through gentle abrasion with a mortar and pestle and soaked in H_2_O for 48 h. Seeds were germinated on damp tissue in controlled growth cabinets (Microclima 1600E, Snijder Scientific) in white light (WL) (cool white fluorescent tubes 400–700 nm, Photosynthetically Active Radiation (PAR) = 70 μmolm^−2^s^−1^ measured at the soil surface, R:FR = 5) in 12 h photoperiods, 20 °C and 70% relative humidity. After 3 days in these conditions, germinated seeds were selected for potting on to a 3:1 mixture of compost (Levingtons F2) and silver sand to a depth of 10 mm. Subsequently, plants were moved to treatment conditions (12 h light:12 h dark cycles, 20 °C and 70% relative humidity). R:FR ratios were set at 5 for high R:FR and 0.05 for low R:FR using supplementary Far Red LEDs (+FR). Supplementary UV-B at 1.5 μmolm^−2^s^−1^ (0.6 W m^−2^) was provided with Philips TL100W/01 narrow band UV-B bulbs. Polycarbonate filters (6 mm thickness) were used to attenuate UV-B for plants grown in -UV-B conditions. See Figure S1a for light spectra. Arabidopsis seeds were surface sterilised with 70% v/v EtOH and 20% v/v Sodium Hypochlorite treatments. After three days stratification in darkness at 4 °C, seeds were germinated following similar protocols to coriander.

For glasshouse experiments, plants were exposed to ambient PAR levels which ranged from 60 to 800 µmolm^−2^s^−1^ throughout the experiment (Figure S1b). A minimum PAR of 165 µmolm^−2^s^−1^ and 16 h photoperiods were maintained using supplementary fluorescent lamps (Plug and Grow compact 200 W). UV-B supplementation was provided using Philips TL100W/01 narrow band UV-B bulbs. Temperature was maintained at 18 °C.

### Image analysis

Morphological data from seedlings and mature plants was extracted from images using FIJI^21^. Hypocotyls were measured from the shoot apical meristem to the shoot-root junction. Petiole lengths were measured from the shoot apex to the base of the leaf blade. Visible leaf area was measured by counting pixels from binarized images of flattened leaf blades.

### Timelapse imaging of elongating hypocotyls

For Time-Lapse Infra Red (IR) photography, a custom built 8 × 8 array of 880 nm IR LEDs were controlled with a 24 h timer. Timelapse images were captured with a Nikon D80 DSLR camera with its IR blocking filter removed, a SIGMA 105 mm macro lens and an IR pass filter (>850 nm) (Zomei, Jiangsu, China) operated with Digicamcontrol software. Images were taken at 60 min intervals. Timelapse start and duration was as specified in the experiment. Hypocotyl lengths from individual images and time lapse image stacks were manually measured from images using FIJI.

### Chlorophyll abundance

Leaf chlorophyll content was determined by the Witham *et al*. method^22^. 100 mg tissue from leaf 2 of 28-day-old plants was snap frozen at −80 °C and homogenised using stainless steel beads and a TissueLyser (Qiagen). Chlorophyll from homogenised tissue was extracted in 80% (v/v) acetone. Absorbances were recorded at 663, 645 and 652 nm with 80% acetone used as a blank.

### Total antioxidant activity

Total antioxidant capacity was recorded using a commercially available kit (Total Antioxidant Capacity Assay kit, Sigma-MAK187). 100 mg of leaf tissue from leaf 3 was frozen at −80 °C and homogenised using stainless steel beads and a TissueLyser (Qiagen). Samples were extracted in 1 ml of ice cold 1 X Phosphate Buffered Saline (PBS) and the supernatant was diluted 1:100 to bring values within range of kit standards. Samples were assayed according to the manufacturer’s protocol, by comparing the absorbances of diluted extracts at 570 nm with Trolox standards. Values were then normalised to tissue fresh weight.

### Detection of flavonol glycosides by thin layer chromatography

Flavonol glycoside extraction and thin layer chromatography were carried out as described previously^23^. 100 mg of leaf tissue was homogenised and extracted in 0.4 ml 80% (v/v) methanol. Samples were incubated for 15 min at 70 °C then centrifuged for 10 min. Supernatants were vacuum-dried at 65 °C and dried pellets dissolved in 1 μl 80% methanol mg^−1^ fresh weight.

1 μl of methanolic extracts were spotted onto HPTLC silica gel 60 glass plates (Millipore). Chromatography was performed in a closed glass tank with a mobile phase of ethyl acetate, formic acid, acetic acid and water (100:26:6:12 v/v). After separation, plates were air dried and flavonols detected by spraying 2 ml of of 1% (w/v) 2,3-dibromopropanal (DPBA) in MeOH 3 times with 5 min between sprayings. This was followed by 2 ml of 5% (w/v) PEG 4000 in MeOH 3 times with 5 min between sprayings. After 15 min, the stained HPTLC plate was visualized under UV (365 nm). Flavonol glycoside-DPBA derivatives fluoresce under UV light. Liquid chromatography-Mass Spectrometry (LC-MS) has been used to profile these flavonol glycosides and assign them to different colours^23^. Here we compared methanolic extracts of coriander against *Arabidopsis thaliana* flavonoid biosynthesis mutants to gain qualitative information about flavonoid content.

### Light Measurements

Data were recorded using Ocean Optics FLAME and USB2000 spectrophotometers and analysed using Ocean Optics Oceanview software and Microsoft Excel.

### Statistical Analyses

SPSS v. 23 (IBM) was used to analyse quantitative data.

### Data Availability

The datasets generated during and/or analysed during the current study are available from the corresponding author on reasonable request.

## Results

### UV-B inhibits shade avoidance responses in coriander

**Figure 1:**
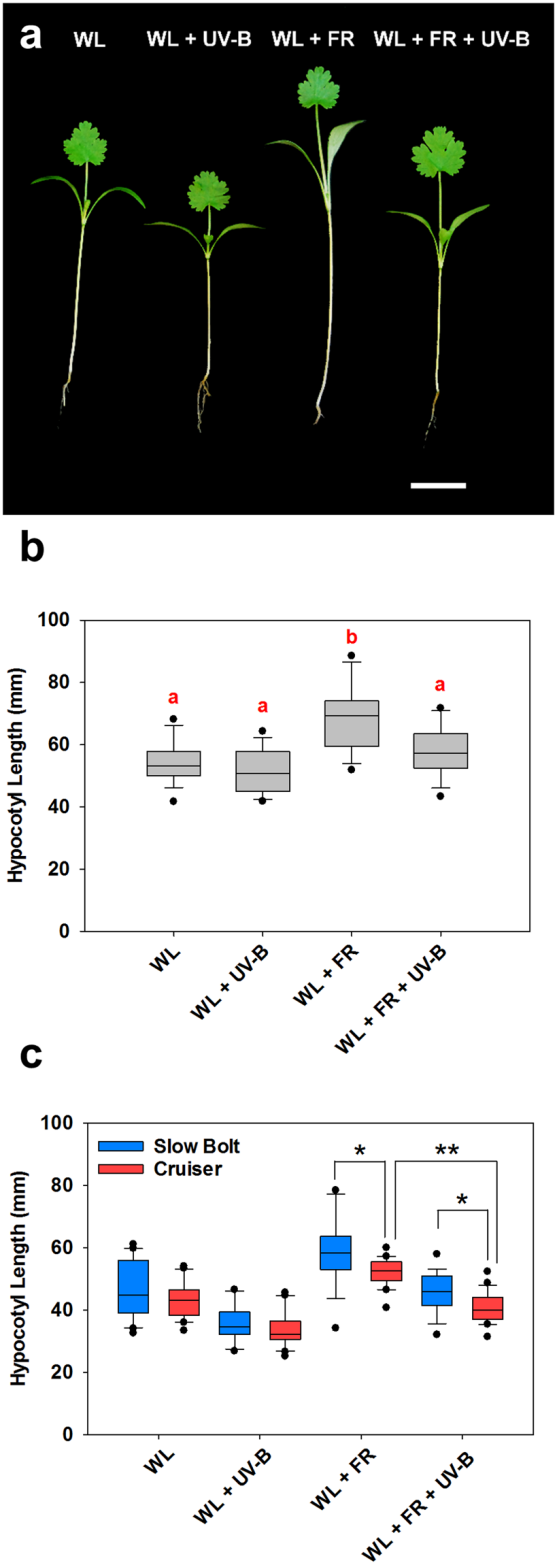
UV-B inhibits hypocotyl elongation in shade-avoiding coriander. (**a**) Coriander (Slow Bolt) seedlings were germinated in WL for 3 days and then placed into WL, WL + UV-B, WL + FR or WL + FR + UV-B conditions as indicated for a further 10 days. Scale bar = 20 mm (**b**) Hypocotyl Lengths (mm) as grown in (**a**) ANOVA (*F*(3,60) = 13.865 *p* < 0.001) *n* = 16. Different red Letters indicate statistically significant differences by Tukey’s post hoc test at p < 0.05. (**c**) Hypocotyl elongation in different coriander cultivars. Slow Bolt (Blue) and Cruiser (Red) were germinated and grown as in (**a**). *n* = 18–21 seedlings. Student’s T-tests were performed to test for differences between cultivars in the indicated conditions. *Indicates significant difference between cultivars at *p* < 0.05. WL + FR (*t*(37) = 2.624, *p* = 0.0126), WL + FR + UV-B (*t*(38) = 2.541, *p* = 0.0153). **Indicates significant difference between WL + FR and WL + FR + UV-B in the Cruiser cultivar at p < 0.05 (*t*(39) = 7.988, *p* < 0.001).

Coriander seedlings were grown in high (5) and low (0.05) R:FR ratio conditions with and without UV-B supplementation (Figs 1a, S1a). Plants grown in low R:FR ratio (+FR) were significantly elongated when compared to plants grown in white light alone (WL), confirming that coriander displays shade avoidance (Fig. 1b)^7^. Low dose UV-B did not result in significantly shorter hypocotyls in a background of WL (Fig. 1b). In low R:FR, however, UV-B-mediated inhibition of hypocotyl elongation was observed (Fig. 1b). These data show that low dose UV-B antagonises shade avoidance in coriander, similarly to Arabidopsis^5^. Interestingly, the magnitude of hypocotyl elongation was cultivar-dependent. Both the ‘Slow Bolt’ and ‘Cruiser’ cultivars displayed enhanced hypocotyl elongation in low R:FR, but absolute lengths for ‘Slow Bolt’ were significantly longer than ‘Cruiser’. UV-B inhibited low R:FR-induced hypocotyl elongation of both cultivars, although the hypocotyl lengths of the ‘Slow Bolt’ cultivar were, again, significantly longer than ‘Cruiser’ (Fig. 1c). Due to its enhanced stem elongation, we selected ‘Slow Bolt’ for further experimentation.

**Figure 2:**
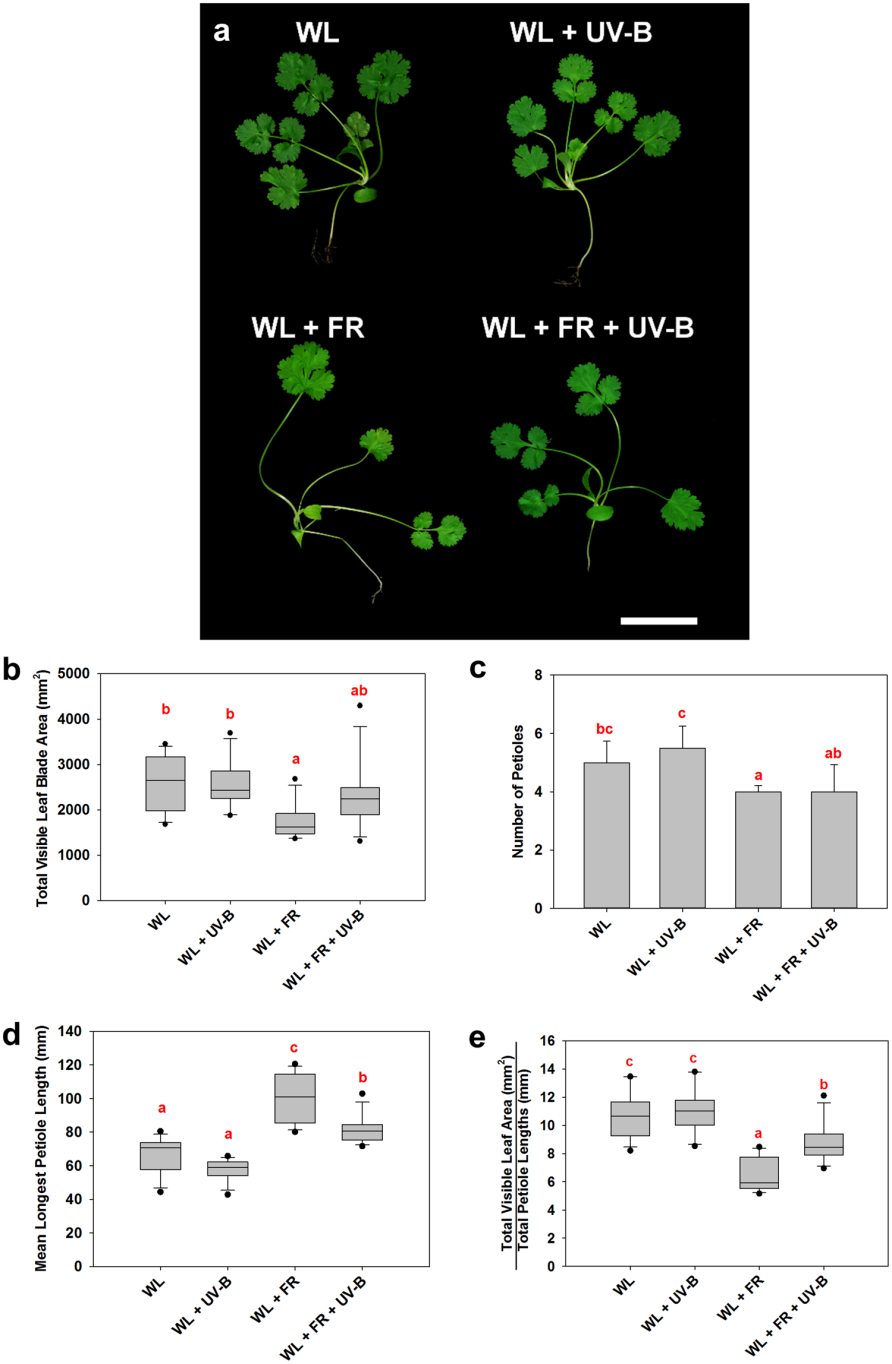
UV-B inhibits petiole elongation but does not significantly increase leaf blade area or petiole number in shade-avoiding coriander. (**a**) 28-day-old plants grown in 12 h photoperiods. Seedlings were germinated in WL for 3 days and then placed into WL, WL + UV-B, WL + FR or WL + FR + UV-B conditions as indicated for a further 25 days. Scale bar = 50 mm. (**b**) Total Visible Leaf Blade Area, ANOVA (*F*(3,44) = 5.696, *p* = 0.002. (**c**) Petiole number, Median + /− 1 S.D., ANOVA (*F*(3,44) = 19.319, *p* < 0.001). (**d**) Mean Petiole length, ANOVA (*F*(3,44) = 32.694, *p* < 0.001 (**e**) Ratio of Total Visible Leaf Area to Total Petiole Length, ANOVA (*F*(3,44) = 25.926, *p* < 0.001. *n* = 12. Different Letters indicate statistically significant differences by Tukey’s post hoc test at p < 0.05.

We next examined the effects of low R:FR and UV-B on adult plants. These were grown in the indicated conditions until they were 28 days old, which is similar to the age at which commercially-grown coriander is transported to customers. The morphology of mature plants is more complex than juveniles as coriander produces multiple petioles with variable leaf phenotypes (Fig. 2a). Shade-avoiding Arabidopsis plants have been shown to produce fewer leaves and smaller leaf blades, as resources are diverted towards stem elongation^24,25^. A complex interaction between low R:FR and UV-B signalling has additionally been reported, with low R:FR promoting leaf expansion in the presence of UV-B^5^. We therefore quantified total visible leaf blade area in coriander. UV-B did not significantly affect this parameter in a background of WL. Low R:FR, however, significantly reduced total visible leaf blade area. In contrast to hypocotyl elongation data (Fig. 1), UV-B did not antagonise this response (Fig. 2b). Total visible leaf blade area could be correlated with petiole number. We therefore compared petiole number between treatments. UV-B did not significantly affect petiole number in a background of WL (Fig. 2c). Low R:FR significantly reduced petiole number which may contribute to the reduced total visible leaf blade area recorded in these plants. This response was not, however, antagonised by UV-B (Fig. 2c).

In low R:FR, UV-B treated plants appeared more compact than untreated controls, which was reflected in the lengths of the longest petioles. UV-B had no effect on petiole length in a background of WL (Fig. 2d). Low R:FR treatment significantly elongated petioles, consistent with shade avoidance^7^. As with hypocotyls, UV-B supplementation antagonised petiole elongation in these conditions (Fig. 2d). The ratio of total visible leaf area/total petiole length was used as a measure of plant compactness. UV-B did not significantly affect plant compactness in a background of WL (Fig. 2e). Low R:FR significantly reduced plant compactness, resulting in more spindly plants (Fig. 2a,e). This response was partially attenuated by UV-B supplementation (Fig. 2e).

Our data show that light quality has significant effects on coriander architecture. Low R:FR drives elongation of hypocotyls and petioles, with both responses inhibited by UV-B. In contrast, low R:FR-mediated reductions in total leaf area and petiole number were not significantly attenuated by UV-B. Nevertheless, plants treated with UV-B in a low R:FR background were still more compact than their untreated controls as demonstrated by the ratio of total visible leaf area to total petiole length (Fig. 2e).

**Figure 3:**
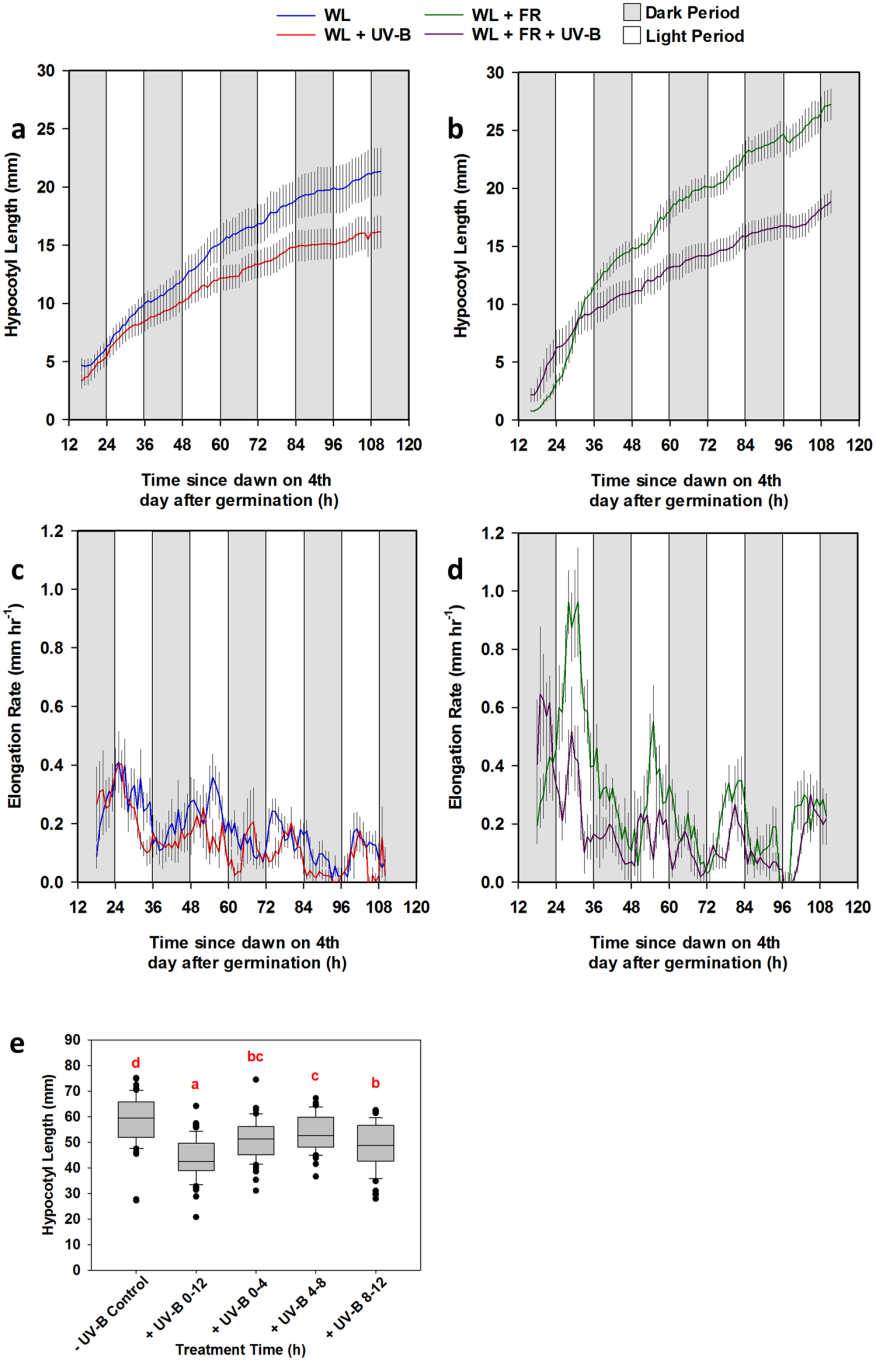
Coriander exhibits rhythmic growth but the timing of UV-B supplementation is unimportant for inhibition of shade avoidance. Seedlings were germinated in WL for 3 days then placed into the indicated conditions for timelapse imaging. (**a**–**d**) Hourly hypocotyl length and growth rate in WL ± UV-B (**a**,**c**) and (WL + FR) ± UV-B (**b**,**d**) n = 8 ± 1 S.E.M. (**e**) Seedlings were germinated in WL for 3 days and then placed into WL + FR and WL + FR supplemented with UV-B at the indicated times for a further 10 days. 0–12 represents a control, whereby UV-B was provided throughout the light period. *n* = 57–61. ANOVA (*F*(4,288) = 25.484, *p* < 0.001) Different Letters indicate statistically significant differences by Tukey’s post hoc test at p < 0.05.

The data described above suggest that UV-B supplementation may be a potential method for increasing the compactness (and therefore aesthetic quality) of densely-grown coriander in commercial settings. The harmful effects of UV-B exposure do, however, present health and safety concerns for workers and continuous UV-B illumination is not economically or environmentally desirable. We therefore questioned whether there was an optimum time of day for UV-B-mediated inhibition of shade avoidance. In short (8 h photoperiod) day- grown Arabidopsis, hypocotyl elongation is greatest at the end of the night, with the phase of maximum elongation growth moving in to the day with longer photoperiods^26^. We applied time lapse infrared imaging to analyse diurnal growth rhythms of coriander grown in 12 h photoperiods. Our data show clear UV-B-mediated inhibition of growth in both white light (Fig. 3a) and low R:FR (Fig. 3b). In all conditions, the period of maximum growth occurred during the light period (Fig. 3c,d). To investigate whether an optimal time of day exists for UV-B-mediated suppression of shade avoidance, we applied a 4 h UV-B treatment at 3 different times of day (1–4 h, 4–8 h, 8–12 h). Our results show that a short (4 h) dose of UV-B is sufficient to significantly inhibit hypocotyl elongation and that the time of day of this treatment has only marginal effects on the magnitude of the response (Fig. 3e). A 4 h UV-B treatment given in the final third of the day inhibited hypocotyl elongation the greatest with a 17% decrease in mean hypocotyl length recorded between treated and control plants. UV-B treatment at the start of the day was the next effective (13% decrease), followed by treatment in the middle of the day (8% decrease). Notably, the 12 h UV-B treatment resulted in the shortest hypocotyls (25% decrease) suggesting that total UV-B dose may be the determining factor in regulating hypocotyl growth inhibition.

### UV-B elevates leaf flavonoid content and antioxidant activity

**Figure 4:**
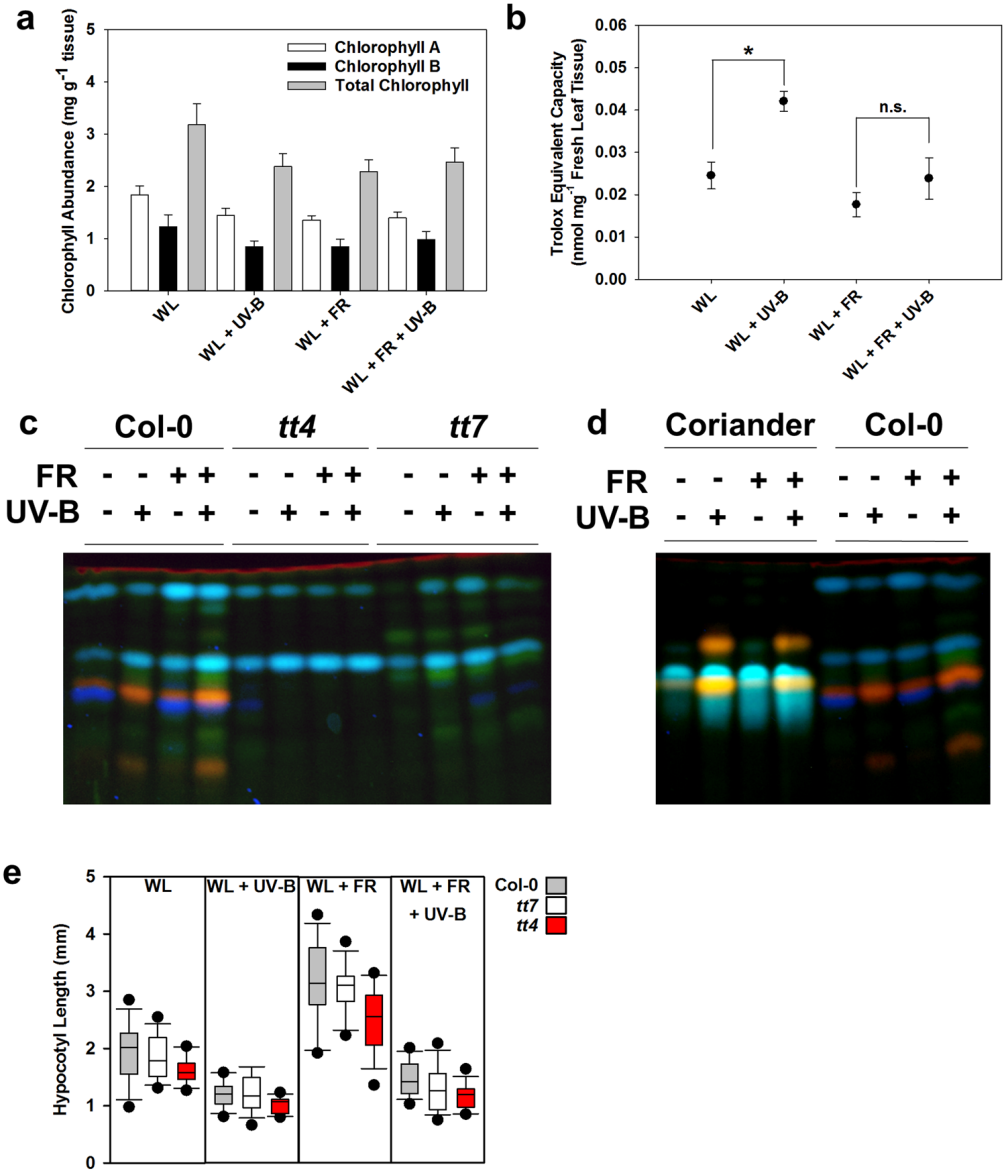
UV-B and low R:FR ratio modulate coriander leaf anti-oxidant capacity and quercetin content. Leaf tissue was sampled from 28-day-old plants grown in 12 h photoperiods. Seedlings were germinated in WL for 3 days and then placed into WL, WL + UV-B, WL + FR and WL + FR + UV-B conditions as indicated for a further 25 days. (**a**) Chlorophyll A and B content in different light conditions. Chlorophyll A, ANOVA (F(3,12) = 2.84, p = 0.083); Chlorophyll B, ANOVA (F(3,12) = 2.84, p = 0.363); Chlorophyll A and B, ANOVA (F(3,12) = 1.918, p = 0.181) *n* = 4. Means ± 1 S.E.M. (**b**) Anti-oxidant activity (in Trolox equivalent nmol mg^−1^ fresh leaf tissue) in UV-B- treated plants (*n* = 8). Data represent means ± 1 S.E.M. In high R:FR, *t*(14) = −4.419, *p* = 0.000584. *statistically significant differences at p < 0.05. (**c**,**d**) Leaf flavonol glycoside accumulation as assayed by thin layer chromatography and derivation with DPBA. Flavonol glycoside derivatives are imaged with UV-illumination (365 nm). Fluorescent Colour key: green = kaempferol derivatives; orange = quercetin derivatives; blue = sinapate derivatives and unknown substances; dark red = chlorophyll. (**e**) Hypocotyl elongation of flavonoid biosynthesis mutants. Plants were grown in long (16 h) photoperiods for 3 days in WL and then 4 days in the indicated light conditions. Two Way ANOVA interaction with genotype and light condition as factors (F(6,166) = 1.480, *p* = 0.188).

A key objective for the herb growing industry is the production of visually appealing plants with dark green leaves. Previous reports have found that light quality can impact chlorophyll abundance^27^ and photosynthetic efficiency^28^, but in our conditions neither R:FR ratio nor UV-B significantly affected chlorophyll content in mature coriander leaves (Fig. 4a).

Low dose UV-B has been hypothesised to act as a ‘eustress’ that activates antioxidant defences prior to the onset of oxidative pressure that can be caused by exposure to high ‘distress’ levels of UV-B^29^. We therefore assayed antioxidant capacity in 28-day-old coriander leaves. WL + UV-B-treated plants displayed significantly greater antioxidant capacity than WL-grown plants (Fig. 4b). Consistent with studies in other species^27^, low R:FR-treated plants had a reduced antioxidant capacity when compared to WL-grown plants. In a background of low R:FR, UV-B did not elevate leaf antioxidant capacity, suggesting that low dose UV-B is not sufficient to counteract the reduction in antioxidant capacity caused by a low R:FR ratio (Fig. 4b).

Flavonols are some of the most abundant flavonoids in plants and act as UV-B filters due to their absorption of UV light in the 280–320 nm region. UV-B induces flavonol accumulation^30^, which impacts on plant flavour^31^ and potentially increases health benefits^32^. We assayed coriander leaves for changes in flavonol glycoside content in response to UV-B using thin layer chromatography and derivation with DPBA as described previously^23^. Arabidopsis mutants, deficient in flavonoid synthesis were included as controls. Under UV-illumination (365 nm), methanolic flavonol glycoside extracts conjugated with DPBA fluoresce as follows: green- kaempferol derivatives; orange- quercetin derivatives; blue- sinapate derivatives and unknown substances; dark red- chlorophyll^23^. In Arabidopsis, CHALCONE SYNTHASE (encoded by the *TT4* gene) catalyses the first step of the flavonoid biosynthesis pathway^20^ and *tt4* mutants clearly lack flavonol glycosides as indicated by the absence of green and orange derivatives when compared to wild-type extracts (Fig. 4c). The FLAVONOID 3′-HYDROXYLASE enzyme (encoded by the *TT7* gene) of the flavonoid biosynthesis pathway catalyses the conversion of dihydrokaempferol to dihydroquercetin^20^. The absence of orange derivatives in *tt7* mutant extracts therefore indicates the absence of quercetin derivatives. In both high- and low R:FR-grown coriander, UV-B predominantly induces the accumulation of quercetin (as indicated by orange fluorescent derivatives) (Fig. 4d).

Flavonoids have been shown to modulate auxin responses in plants. In particular, quercetin has been shown to inhibit polar auxin transport *in planta* ^33^. UV-B-induced flavonoid production may therefore, in part, contribute to the inhibition of shade avoidance in low R:FR. We therefore analysed hypocotyl elongation in *tt4* and *tt7* mutants grown in high and low R:FR with and without UV-B supplementation. No statistically significant differences were observed between wild-type and mutant plants, suggesting that UV-B-induced flavonoid production does not contribute to UV-B-mediated inhibition of shade avoidance.

### UV-B supplementation inhibits stem elongation in the glasshouse

**Figure 5:**
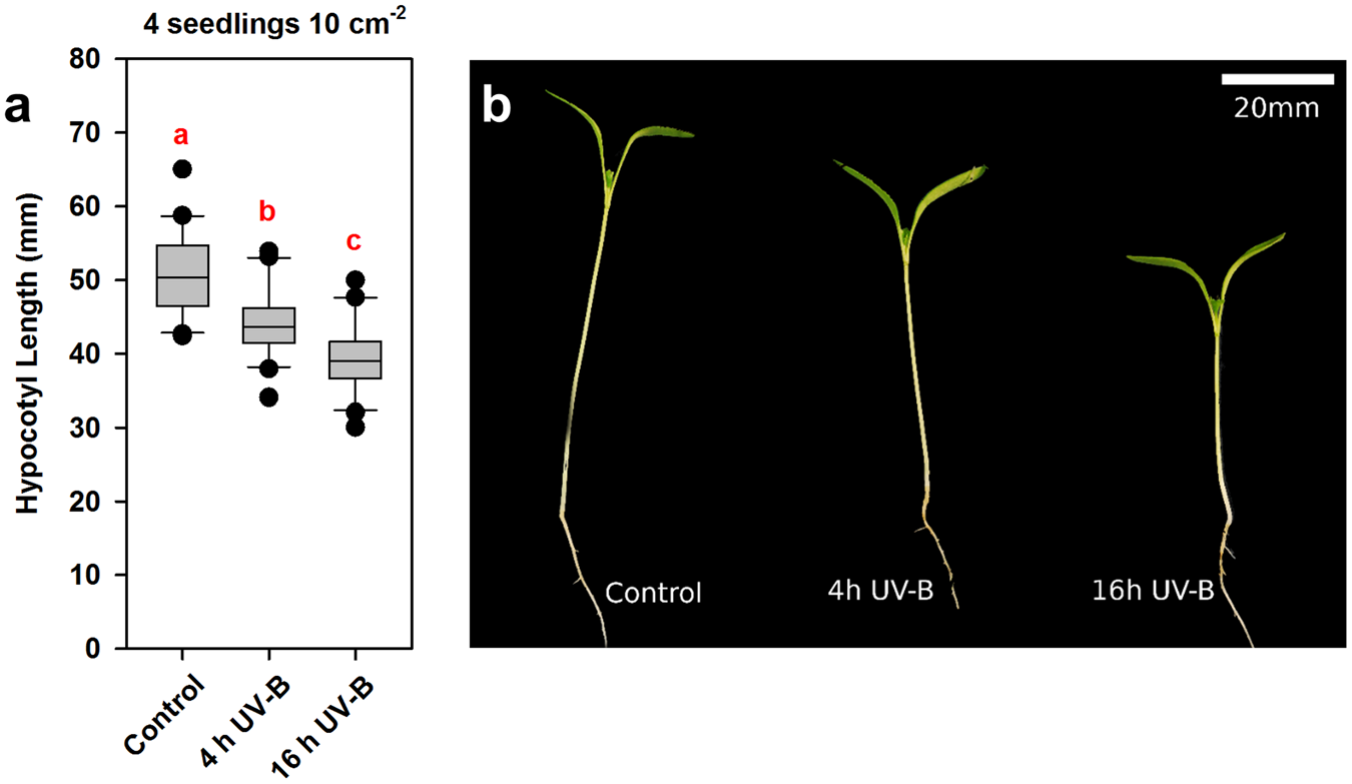
UV-B supplementation inhibits coriander hypocotyl elongation in the glasshouse. Seedlings were grown for 10 days in 16 h photoperiods maintained by supplemental light. Narrow band UV-B was provided for either the entire photoperiod (16 h) or for 4 h at the middle of the day. (**a**) Hypocotyl lengths of seedlings grown at a density of 4 per 10 cm^−2^, n = 20, ANOVA (*F*(2,57) = 23.106, *p* < 0.001). Different Letters indicate statistically significant differences by Tukey’s post hoc test at *p* < 0.05. (**b**) Photograph of representative seedlings as seen in (**a**).

Laboratory experiments are often constrained by the maximum light levels produced by commercial growth cabinets. To assess the effectiveness of UV-B supplementation as a means for plant architecture manipulation in commercial horticulture, we performed glasshouse experiments with sunlight levels of PAR (Figs 5, S1b). A daily 4 h UV-B treatment (1.5 µmolm^−2^s^−1^) given in the middle of the day sucessfuly inhibited hypocotyl elongation in 10-day-old seedlings. Greater hypocotyl elongation was observed when UV-B treatment was applied throughout the photoperiod (16 h) (Fig. 5). When plants were grown for 32 days, 4 h and 16 h UV-B treatments were similarly effective at inhibiting hypocotyl elongation (Figure S2a). Inhibition of petiole elongation was, however, only observed with continuous (16 h) UV-B treatment.

## Discussion

Consistent with studies in Arabidopsis, we found that low dose UV-B (equivalent to a summer afternoon in the UK^34^) antagonised low R:FR-induced stem elongation in coriander^5^ (Figs 1, 2). The shade avoiding nature of coriander may contribute to its spindly appearance when grown commercially in dense stands. In a background of WL (PAR = 70 μmolm^−2^s^−1^), UV-B did not significantly inhibit hypocotyl elongation. It is, however, possible that UV-B may suppress stem growth in a low PAR environment where blue and red light are simultaneously depleted^35^. Hypocotyl elongation responses to light quality treatments differed significantly between cultivars (Fig. 1c). The ‘Cruiser’ cultivar is a compact variety and while it does not elongate as much as ‘Slow Bolt’ in response to low R:FR, its elongation is still inhibited by UV-B supplementation. Phytochrome B (phyB) promotes shoot branching by suppressing auxin signalling in stems. Inactivation of phyB in low R:FR therefore reduces branching, establishing apical dominance^36^. UV-B did not significantly affect coriander branching or leaf blade expansion in our conditions, but increased plant compactness, primarily through inhibition of petiole elongation (Fig. 2). The plant growth hormone auxin is a key regulator of stem elongation, with an established role in driving shade avoidance^7^. UV-B has previously been reported to suppress auxin biosynthesis and signalling in Arabidopsis shade avoidance, thereby reducing plant stem elongation and promoting a compact phenotype^5^. The similarities in architectural response to low R:FR and UV-B displayed by Arabidopsis and coriander suggest that similar signalling mechanisms may exist in both species.

UV-B levels vary diurnally in natural environments^34^. In Arabidopsis, transcriptional responses to UV-B have been shown to be gated by the circadian clock in free running conditions and vary depending on the time of day in light-dark cycles^37,38^. Coriander displayed maximum growth during the day in 12 hour photoperiods, consistent with Arabidopsis grown in longer photoperiods (Fig. 3a–d). In our experiments, time of day had little impact on UV-B-mediated inhibition of hypocotyl elongation (Fig. 3e). We predicted that UV-B treatment would maximally inhibit elongation growth in the afternoon, when shade is most effective at promoting hypocotyl elongation in diurnal cycles^39^ and sunflecks are most effective at inhibiting shade avoidance within canopies^40^. Our data may suggest that, unlike many reported UV-B responses, UV-B-mediated hypocotyl growth inhibition is not gated by the circadian clock. Data may also reflect species-specific differences in circadian regulation or photomorphogenesis between Arabidopsis and coriander. Alternatively, circadian gating of UV-B-mediated hypocotyl growth inhibition may negate opposing rhythms of UV-B responsiveness, thereby ensuring uniform UV-B-mediated growth inhibition throughout the day and preserving rhythmic growth^26^.

Low R:FR ratio can reduce leaf chlorophyll content^27^. A similar response has been observed in coriander in response to high dose narrowband UV-B and likely results from damage to the photosynthetic apparatus^19^. Given the documented repair of the Earth’s ozone layer^41^, greater research effort is now being focussed on the regulatory effects of low-dose, non-harmful UV-B radiation, which has been implicated in improving photosynthetic efficiency^28^. We found no effect of R:FR or UV-B on leaf chlorophyll content (Fig. 4a), suggesting that coriander chlorophyll accumulation may not be as sensitive to light quality as in other species.

Following UV-B perception, plants activate antioxidant defences to alleviate oxidative pressure and minimise DNA damage via a UVR8-COP1-HY5 signalling pathway^1–3^. This includes increased synthesis of flavonoids, including the ‘effective antioxidant’ quercetin^42^. We therefore assayed leaf tissue for total antioxidant capacity after low dose UV-B irradiation (Fig. 4b). Consistent with other studies^43^, low dose UV-B significantly increased antioxidant capacity in plants grown in white light. The same UV-B treatment given to coriander grown in low R:FR was, however, ineffective. We found that plants grown in low R:FR had reduced antioxidant capacity compared to white light- grown controls, consistent with findings in other species^27^. It is possible that this represents the diversion of resources from defence towards elongation^44^. Alternatively, as low R:FR ratio is a signal of impending shade, plants may minimise photoprotective mechanisms in these conditions. It is intriguing that UV-B, a component of direct sunlight, did not significantly elevate antioxidant capacity in low R:FR ratio and suggests that low R:FR ratio may block UV-B-mediated activation of antioxidant defences. However, in natural environments, plants would only perceive sunlight levels of UV-B in combination with very low R:FR when emerging from a canopy.

Due to their absorption of UV light in the 280–320 nm region and the hypersensitivity of flavonoid biosynthesis mutants to UV-B radiation^45^, flavonoids are regarded as sunscreening compounds in Arabidopsis. UV-B induces their accumulation^30^ and CHALCONE SYNTHASE, an enzyme that catalyses the conversion of coumaroyl-CoA and malonyl-CoA in the flavonoid biosynthesis pathway^20,46^, is a key marker for plant UV-B signalling^47^. Our analysis of flavonoid levels suggested that UV-B mainly induces the accumulation of quercetin in coriander (Fig. 4c). An increase in quercetin levels following UV-B treatment has previously been reported in unrelated taxa such as petunia^48^, *Brassica napus* ^49^ and apple^50^. Like other flavonoids, quercetin has UV-B absorptive properties, but it also acts as an antioxidant, which is likely due to multiple hydroxy groups in the A, B and C aromatic rings^51^. Intriguingly, in a background of low R:FR, we observed a clear increase in quercetin but no significant increase in total antioxidant capacity (Fig. 4b,c). It is possible that this reflects low R:FR-mediated supression of antioxidant activity, as described above. Despite conveying potential health benefits, quercetin, like other flavonoids, is associated with bitter flavours^52^. Growers will therefore need to consider the potential flavour alterations caused by UV-B supplementation alongside aesthetic improvements.

In addition to their antioxidant properties, flavonoids have been shown to negatively regulate auxin transport, affecting shoot branching, lateral root formation and root gravitropism^33^. In Arabidopsis, low R:FR-induced auxin biosynthesis and transport are central to shade avoidance^53^. Low R:FR increases expression and re-localisation of the auxin efflux regulator PIN-FORMED 3 (PIN3) to direct auxin to the hypocotyl epidermis, promoting cell elongation^54^. We therefore speculated that the flavonoid deficient *tt4* and *tt7* mutants may display impaired UV-B-mediated inhibition of shade avoidance. These mutants behaved similarly to wild-type controls, suggesting that UV-B-mediated flavonoid production is not a key component of shade avoidance inhibition.

Collectively, our results suggest that the inclusion of UV-B into commercial growth regimes may give appreciable benefits to coriander architecture and phytonutrient content. Although a daily 4 h UV-B treatment was sufficient to elicit a significant suppression of hypocotyl elongation in both growth cabinet and glasshouse settings (Figs 3e, 5, S2), our experiments indicate that timing of the UV-B dose is relatively unimportant (Fig. 3e). An additional consideration in a glasshouse setting is that different photon irradiances of UV-B may need to be applied to maintain the UV-B:PAR ratio, as light quantity changes throughout the day. Future applications are likely to utilise advancements in UV-B LED technology^55^, although a more cost-effective approach may involve the construction of greenhouses from materials with higher UV-B transmission^56^. It will be of interest to see if UV-B transparent materials can improve the architecture, flavour and phytonutrient content of glasshouse- grown coriander in an energy- and cost-effective manner.

## Electronic supplementary material

Supplementary Information
