# Supplementary material for: UV-B antagonises shade avoidance and increases levels of the flavonoid quercetin in coriander (Coriandrum sativum)
Source: Sci Rep. 2017 Dec 19;7:17758. doi: 10.1038/s41598-017-18073-8 (PMC5736551; doi:10.1038/s41598-017-18073-8)
Supplement: Supplementary file 1 — Supplementary Information [file 41598_2017_18073_MOESM1_ESM.pdf]

Supplementary information: UV-B antagonises shade avoidance and increases levels of the flavonoid quercetin in coriander (*Coriandrum sativum*)

Fraser DP, Sharma A, Fletcher T, Budge S, Moncrieff C, Dodd AN and Franklin KA

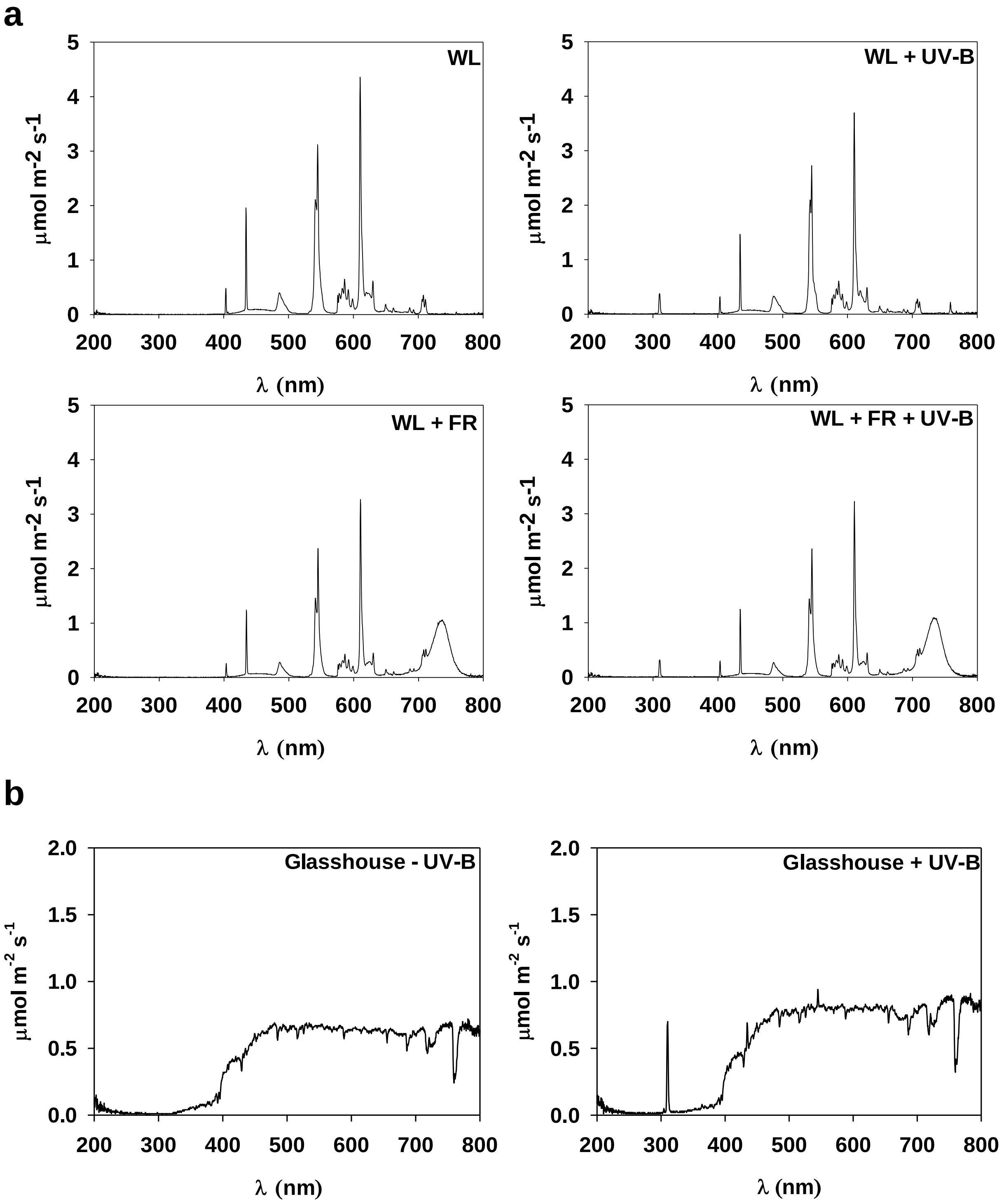

Figure S1 Light spectra from experimental conditions. **(a)** Growth cabinets. 70 µmol m^{-2} s^{-1} of white light supplied with fluorescent bulbs was supplemented with far red LEDs to achieve a R:FR of 0.05. UV-B was supplemented at 1.5 µmol m^{-2} s^{-1} using narrow band fluorescent bulbs. **(b)** Glasshouse. Plants were exposed to ambient light levels typical of spring in Bristol. UV-B was supplemented at 1.5 µmol m^{-2} s^{-1} using narrow band bulbs. 16 h photoperiods were maintained using white fluorescent bulbs.

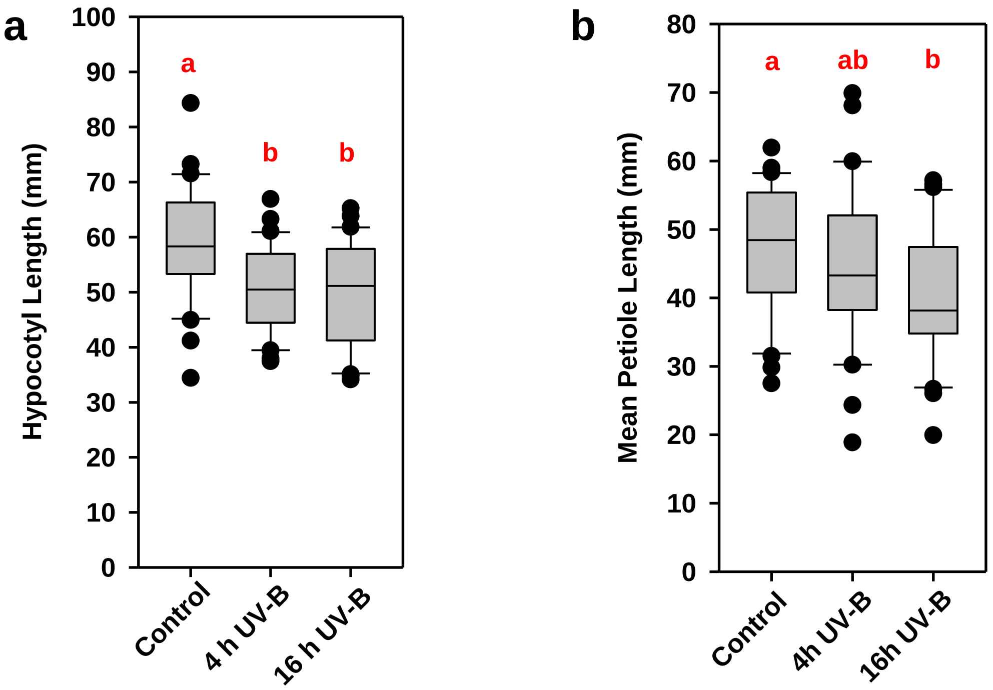

Figure S2 UV-B inhibits mean petiole elongation in mature coriander grown at high density (4 seedlings 10 cm^{-2}) in the glasshouse**.** Coriander was grown for 32 days with 16 h photoperiods maintained by supplementary lighting. UV-B was provided by narrow band UV-B bulbs for either the entire photoperiod (16 h) or for 4 h at the middle of the day. Plotted are morphological data from 32 day old plants **(a)** Hypocotyl Lengths, ANOVA ($F(2,87) = 8.551$, $p < 0.001$). **(b)** Mean Petiole Length ANOVA ($F(2,87) = 4.015$, $p = 0.021$). N = 30. N.S. = no significant difference at $p = 0.05$. Different red letters indicate statistically significant differences at $p < 0.05$.
